# Association of Lipoproteins with Neutrophil Extracellular Traps in Patients with Abdominal Aortic Aneurysm

**DOI:** 10.3390/biomedicines10020217

**Published:** 2022-01-20

**Authors:** Annika Brandau, Nahla Ibrahim, Johannes Klopf, Hubert Hayden, Maria Ozsvar-Kozma, Taras Afonyushkin, Sonja Bleichert, Lukas Fuchs, Viktoria Watzinger, Verena Nairz, Emely Manville, Veronika Kessler, Herbert Stangl, Wolf Eilenberg, Christoph Neumayer, Christine Brostjan

**Affiliations:** 1 Division of Vascular Surgery, Department of General Surgery, Medical University of Vienna, Vienna General Hospital, 1090 Vienna, Austria; AnnikaBrandau@gmx.de (A.B.); nahla.ibrahim@nyu.edu (N.I.); johannes.klopf@meduniwien.ac.at (J.K.); hubert.hayden@meduniwien.ac.at (H.H.); sonja.bleichert@meduniwien.ac.at (S.B.); n1248453@students.meduniwien.ac.at (L.F.); vickywatzi@gmail.com (V.W.); verenanairz@gmail.com (V.N.); emely.manville@hotmail.com (E.M.); veronika.kessler@gmail.com (V.K.); wolf.eilenberg@meduniwien.ac.at (W.E.); christoph.neumayer@meduniwien.ac.at (C.N.); 2 Department of Laboratory Medicine, Medical University of Vienna, Vienna General Hospital, 1090 Vienna, Austria; maria.ozsvarkozma@meduniwien.ac.at (M.O.-K.); taras.afonyushkin@meduniwien.ac.at (T.A.); 3 Center for Pathobiochemistry and Genetics, Department of Medical Chemistry, Medical University of Vienna, 1090 Vienna, Austria; herbert.stangl@meduniwien.ac.at

**Keywords:** abdominal aortic aneurysm, citrullinated histones, lipoproteins, neutrophil extracellular traps, peripheral arterial occlusive disease

## Abstract

Neutrophil extracellular traps (NETs) are DNA–protein structures released by neutrophils in response to various stimuli, including oxidized, low-density lipoprotein (oxLDL). Accumulating evidence suggests a role for NETs in the pathogenesis of abdominal aortic aneurysm (AAA). In this study, we investigated the potential association of lipoprotein particles and NETs in AAA in comparison to non-AAA control groups. The concentrations of neutrophil myeloperoxidase (MPO), the NET parameters citrullinated histone H3 (citH3) and circulating cell-free DNA (cfDNA), as well as of blood lipids were determined in plasma or serum of patients with AAA (*n* = 40), peripheral artery occlusive disease (PAD; *n* = 40) and healthy donors (*n* = 29). A sandwich ELISA detecting oxidized phosphatidylcholine in association with apolipoprotein B-100 (oxPL/apoB) was applied to measure oxidized phospholipids in circulation. The effect of lipoparticles on NET formation was tested using a DNA release assay with isolated human neutrophils. Plasma MPO, citH3 and cfDNA levels were significantly increased in AAA patients in comparison to healthy donors and PAD patients. Plasma concentrations of citH3 positively correlated with serum oxPL/apoB in AAA patients. In functional in vitro assays, the addition of oxLDL induced NET formation in pre-stimulated neutrophils. In conclusion, our data suggest a promoting role of oxLDL on NET formation in AAA patients.

## 1. Introduction

Abdominal aortic aneurysm (AAA) is a precarious condition, as patients are often asymptomatic, while the risk of rupture remains a constant threat and leads to death in 80% of cases [1]. Therefore, it is vital to further improve our knowledge on the pathogenesis of this aortic dilatation, so that future therapies can have a causal mode of action and be effective before surgery is indicated.

It is known that immune cells, particularly neutrophils, accumulate in the intraluminal thrombus and adventitia of the AAA and contribute to the progression of disease [2]. Neutrophils release proteolytic enzymes and cause oxidative stress, thereby promoting the degradation of elastin fibers and the death of smooth muscle cells in the aortic wall [3,4].

Activated neutrophils can form neutrophil extracellular traps (NETs), which are part of the innate immune response in pathogen defense [5]. They are also implicated in disease states of chronic inflammation such as atherosclerosis and AAA [6,7]. The backbone of NETs is the expelled, negatively charged nuclear DNA (chromatin). Due to the web-like structure, NETs function in the containment of pathogens. For the release of chromatin, DNA has to be decondensed, which is promoted by the modification of histones, such as citrullination via peptidylarginine deiminase 4 [8,9]. Hence, citrullinated histone H3 (citH3) is considered a marker of NET formation. Moreover, NETs consist of many antimicrobial substances such as neutrophil elastase, myeloperoxidase (MPO) and other neutrophil proteins that are able to degrade virulence factors and kill bacteria [10,11].

Since NETs are not only involved beneficially in the antimicrobial defense but also adversely contribute to the pathogenesis of various disorders, in particular cardiovascular diseases (CVDs), they have been investigated in patients and preclinical disease models of CVDs [12,13]. NETs and associated circulating blood components were found to indicate severe coronary atherosclerosis in a prothrombotic state [14] as well as the extent and prognosis of myocardial infarction [15,16]. Further studies revealed that NETs are also involved in the pathomechanism of abdominal aortic aneurysms; NET inhibition in a murine AAA model suggested an essential role of NETs in the initial disease phase and identified IL-1β as an inducer of NET formation [17]. NETs were shown to promote the activation of plasmacytoid dendritic cells and the production of type I interferon, which stimulated AAA development by an increased inflammatory response [18]. In the analysis of human AAA samples, the intraluminal thrombus and also the adventitia were found to be enriched with NETs [19]. We have recently reported on elevated levels of citH3 in the tissue and plasma of AAA patients, which showed considerable potential as a biomarker and target to inhibit the progression of AAAs [20].

In cardiovascular diseases, different triggers of NET release have been identified [12]; elevated shear stress in arteries [21] and cholesterol crystals stimulate neutrophils to release NETs [22]. Activated platelets are able to induce NET formation and in turn, NETs may activate platelets and promote coagulation [23,24]. In vitro, NETs can be triggered by various stimuli, of which the protein kinase C activator phorbol 12-myristate 13-acetate (PMA), the calcium ionophore A23187 and the endotoxin lipopolysaccharide (LPS) are commonly applied [25,26]. Among the various NET stimuli, the effect of lipoprotein particles on NET formation is of particular interest with respect to atherosclerosis and CVD. Awasthi et al. reported that oxidized, low-density lipoprotein (oxLDL) stimulates the formation of NETs via the TLR–PKC–IRAK–MAPK pathway and the activation of NADPH-oxidase 2 [27]. More recently, Obama et al. showed that oxLDL enhances the PMA-induced NET formation of HL-60 derived neutrophils when cells are pre-incubated with the stimulus, then washed and subsequently exposed to oxLDL [28]. However, in vivo evidence for an association of lipoprotein levels with NET factors in CVD is currently still missing.

In our study, we thus aimed to compare the blood levels of NET and lipid parameters in patients with distinct forms of CVD, i.e., AAA versus peripheral artery occlusive disease (PAD), and in healthy donors. We hypothesized that circulating NET markers might be particularly high in the AAA group and correlate with blood lipid levels, thus indicating a potential in vivo link in this disease. Furthermore, we aimed to test in vitro whether the cardiovascular-harmful oxLDL and the cardiovascular-protective, high-density lipoprotein (HDL) would exert antagonistic effects on NET formation to potentially corroborate in vivo findings.

## 2. Materials and Methods

### 2.1. Study Design

The clinical study was approved by the Ethics Committee of the Medical University of Vienna (no. 1729/2014) and conducted according to the principles of the Declaration of Helsinki (and current amendments). Participants for the three study cohorts were recruited among Vascular Surgery, Visceral Surgery, Ophthalmology and Urology patients at the Medical University of Vienna, Vienna General Hospital and included 40 AAA patients, 40 PAD patients and 29 healthy controls (Table 1). All gave informed written consent to participate in the study.

Inclusion criterion for AAA patients was the AAA diagnosis with a maximum abdominal aortic diameter of ≥3 cm. Conversely, PAD patients with a confirmed hospital disease record were recruited. The healthy cohort was defined as unaffected by cardiovascular burden regarding clinically manifest disease such as myocardial infarction, coronary heart disease, stents and coronary artery bypass grafts (Table 2).

The exclusion criteria for all three cohorts comprised organ transplantation, recent cancer and/or chemotherapy in the past year, autoimmune disease and chronic hematological disease. For the cohorts of AAA patients and healthy controls, the presence of PAD was excluded. Aneurysm morphology of AAA patients was determined using computed tomography angiography (CTA) scans, while healthy controls underwent an ultrasound examination to exclude the possibility of AAA. The PAD cohort had a pelvis/leg CTA scan or comparable imaging in the recent past, which attested to them being negative for AAA.

The demographics of all participants were collected via a structured questionnaire. While AAA patients and healthy controls were only group-matched regarding age and sex, a one-to-one matching was performed for the AAA and PAD patients based on age (±3 years), sex and occurrence of any previous cardiovascular events (myocardial infarction, coronary heart disease, stents or coronary artery bypass grafts).

### 2.2. Blood Sampling

Peripheral venous blood was collected from all participants and supplied with an anticoagulant mix of citrate, theophylline, adenosine and dipyridamole (CTAD) for plasma preparation within 60 min. The CTAD blood sample was centrifuged twice for 10 min at 4 °C (at 1000× *g*, then 10,000× *g*) to obtain platelet-free plasma. Hemolytic samples were discarded. Additionally, serum was prepared after 1–2 h of clotting time and was centrifuged once for 10 min at 1000× *g* at room temperature. The CTAD plasma and serum samples were stored in aliquots at −80 °C until further testing.

### 2.3. Measurement of Blood Parameters via ELISA

An enzyme-linked immunosorbent assay (ELISA) was used to determine the concentrations of citH3, MPO and oxidized phosphatidylcholine in association with apolipoprotein B-100 (oxPL/apoB) as a measure of oxidized phospholipids and a surrogate of oxLDL in blood samples. The MPO plasma concentration was quantified with the Human Myeloperoxidase Quantikine ELISA Kit (R&D Systems Inc., Minneapolis, MN, USA). To test the citH3 plasma levels, a previously published ELISA protocol was applied [29]. In brief, 96-well plates were coated overnight at 4 °C with an anti-histone antibody (Cell Death Detection ELISA; Roche, Basel, Switzerland). Plasma samples and internal calibrators were applied in duplicates. Detection was performed using an anti-H3Cit antibody (ab5103, Abcam, Cambridge, UK) and an anti-rabbit horseradish peroxidase conjugate (7074S, Cell Signaling, Danvers, MA, USA). The 3,3′,5,5′-tetramethylbenzidine substrate reaction was stopped by acidification and measured at 450 nm in a Varioskan Flash Multimode Reader (Thermo Fisher Scientific Inc., Waltham, MA, USA) with the correction wavelength set to 540 nm.

For the quantification of oxPL/apoB, a 96-well plate (BRAND, immunoGrade, Thermo Fisher Scientific) was coated overnight at 4 °C with the MB47 antibody (Avanti Polar Lipids, Alabaster, AL, USA) directed against apolipoprotein B-100. The serum samples and internal calibrators were then applied in triplicates. The E06 biotinylated monoclonal mouse antibody (Avanti Polar Lipids) was added for the detection of oxidation-specific phosphatidylcholine epitopes on phospholipids. Bound antibody was measured with NeutrAvidin conjugated to alkaline phosphatase (Thermo Fisher Scientific) and Lumi-Phos Plus substrate (Lumigen, Southfield, MI, USA) in a BioTec Synergy 2 plate reader (Thermo Fisher Scientific). A more detailed protocol was previously published [30,31]. Of note, outliers (deviation by ≥25% from both other values) were eliminated from the triplicate measurements.

The routine serum lipid profile and other clinical blood parameters were assessed by the Vienna General Hospital central laboratory. The differential blood count was determined using a Sysmex hematology analyzer (Sysmex Corp., Kobe, Japan).

### 2.4. Detection of Circulating Cell-Free Nuclear DNA by Polymerase Chain Reaction

DNA from 190 µL of citrate plasma was isolated with a DNeasy Blood and Tissue Kit (Qiagen, Venlo, The Netherlands). Levels of circulating cell-free nuclear DNA (cfDNA) were assessed by means of quantitative polymerase chain reaction (qPCR) with a GoTaq^®^ qPCR Master Mix Kit (Promega, Madison, WI, USA) and 200 nM primers amplifying the repetitive nuclear Arthrobacter luteus Alu element (forward primer: 5′-TCA CGC CTG TAA TCC CAG CA-3′, reverse primer: 5′-GTA TTT TTA GTA GAG ACG GGG TTT C-3′). For normalization, an internal calibrator (prepared from plasma of 20 AAA patients) was applied to each qPCR plate, and its DNA content was set to 1.

### 2.5. Lipoprotein Isolation and Modification

HDL particles were isolated from plasma, obtained from normo-lipidemic healthy volunteers, using sequential flotation ultracentrifugation [32]. Blood donations were approved by the Ethics Committee of the Medical University of Vienna (no. 1414/2016). Low-density lipoprotein (LDL) was purchased from Sigma Merck (LP2-2 mg), and oxLDL was prepared with CuSO_4_ and subsequent dialysis as previously described [33].

### 2.6. DNA Release Assay

Neutrophils were isolated from peripheral blood of healthy volunteers anticoagulated with ethylenediaminetetraacetic acid. Blood was layered on a gradient of Histopaque (1077 and 1119, Sigma-Aldrich/ Merck KGaA, Darmstadt, Germany) as previously described [34]. The purity of isolated neutrophils was determined using a Sysmex hematology analyzer (Sysmex Corp., Kobe, Japan) and consistently ranged >95%. A total of 1 × 10^5^ cells in 100 µL of Hank’s balanced salt solution (HBSS) with 0.5% fetal calf serum was seeded in a 96-well plate and then stimulated with 5 µM of A23187 for 30 min. One sample was left unstimulated as a control. The plate was then centrifuged twice at 800× *g* for 5 min at room temperature to remove the supernatant and wash the cells with HBSS before adding 100 µg/mL oxLDL, native LDL (natLDL) or HDL to the primed cells in a total volume of 300 µL of HBSS with 0.5% fetal calf serum. To quantify the DNA release, 5 µM of SYTOX Green (Thermo Fisher Scientific) was added and fluorescence was measured after 90 min at excitation/emission of 485/520 nm using the VarioSkan plate reader (Thermo Fisher Scientific Inc., Waltham, MA, USA).

### 2.7. Statistical Analysis

Statistics were evaluated with SPSS 27.0 software (IBM, Armonk, NY, USA). The significance level was set at *p* < 0.05. For metric data, median values and interquartile range (IQR) were calculated. Non-parametric tests were used to evaluate parameter correlations (Spearman r) and differences between the study groups (Mann–Whitney U test). Categorical variables were interpreted with the help of contingency tables and the Chi-square test or Fisher’s exact test for expected frequencies < 5.

## 3. Results

### 3.1. AAA and PAD Patients Have a Comparable Profile of Comorbidities and Medication While Healthy Controls Differ Significantly

The AAA and PAD cohorts, as well as the healthy donors, showed a comparable distribution (Table 1) regarding age (median 67–73 years), sex (80–90% male) and body mass index (median 25.9–27.3). The healthy controls (H) had a significantly lower percentage of current or past smokers compared to the two CVD cohorts (H: 69%, PAD: 93% and AAA: 93%). Furthermore, the AAA and PAD groups exhibited a similar profile of cardiovascular comorbidities (Table 2) including hypertension (90–93%), dyslipidemia (88–93%), coronary heart disease (43–50%) and myocardial infarction (23–33%). Additionally, diabetes mellitus, nephropathy and chronic obstructive pulmonary disease occurred at a similar frequency in those two cohorts. In contrast, the healthy controls showed a significantly lower prevalence for most of these comorbidities. In line with the recorded comorbidities, the matching medication was more frequently given to AAA and PAD patients in comparison to the healthy cohort; more than 80% of the AAA and PAD patients received anti-hypertensive, anti-platelet and lipid-lowering therapy as opposed to the healthy blood donors, of whom only 52% received anti-hypertensive therapy and merely 17% took anti-platelet or lipid-lowering drugs (Table 3).

### 3.2. Neutrophil and NET Parameters Are Particularly Elevated in Blood of AAA Patients

Hospital routine analysis (Table 4) revealed that the previously reported AAA biomarker D-dimer was highest in AAA patients (median levels in H: 0.38 µg/mL, PAD: 0.75 µg/mL, AAA: 1.26 µg/mL), i.e., was significantly elevated compared to PAD patients (*p* = 0.008) and healthy controls (*p* < 0.001). The inflammation marker CRP, however, did not differ significantly between the three groups (0.21–0.27 mg/dL).

In differential blood count, neutrophils were found to be significantly elevated in the PAD (*p* < 0.001) and AAA patients (*p* = 0.016) in comparison to the healthy controls (median levels in H: 3.0 × 10^3^/µL, PAD: 4.5 × 10^3^/µL, AAA: 4.0 × 10^3^/µL). Of note, the PAD patients also had a significantly higher neutrophil count than the AAA cohort (*p* = 0.023). For a subset of patients (three individuals per group), neutrophil identification and quantitation in whole blood was confirmed using flow cytometry based on CD62L+ CD66b+ CD16+ cell staining, as we have previously published [35]. A high degree of correlation was observed between neutrophil levels in differential blood count and in flow cytometric analysis (Spearman coefficient *r* = 0.983, *p* < 0.001).

**Figure 1:**
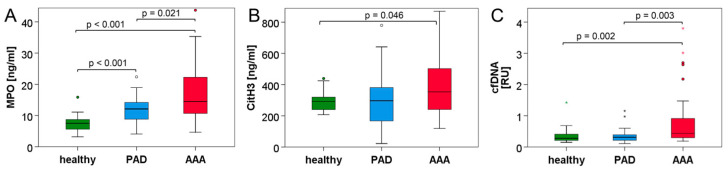
Neutrophil activation and NET parameters are elevated in the blood of AAA patients. Plasma concentrations of myeloperoxidase (**A**), citrullinated histone H3 (**B**) and cell-free DNA (**C**) were determined by ELISA (**A**,**B**) or qPCR (**C**) in the blood of healthy donors and patients with PAD or AAA. Statistical significance was determined by Mann–Whitney U test. Of note, in (**A**,**B**), several outliers/extreme values are not shown for better plot resolution.

In order to detect neutrophil activation and NET formation in cardiovascular disease, plasma samples of the three groups were further investigated for MPO, citH3 and circulating cfDNA (Figure 1, Table 5). MPO differed significantly between all three collectives and showed the highest levels in the AAA group (median H: 7.5 ng/mL, PAD: 12.1 ng/mL, AAA: 14.5 ng/mL). Comparably, plasma citH3 was significantly elevated in AAA patients versus healthy controls (*p* = 0.046) and showed a statistical trend to be elevated in AAA compared to the PAD group (*p* = 0.070). However, circulating citH3 was not increased in PAD patients in comparison to the healthy blood donors (median H: 292 ng/mL, PAD: 297 ng/mL, AAA: 353 ng/mL). Circulating cfDNA was measured in a subset of participants and was also found to be significantly higher in AAA than PAD patients (*p* = 0.003) or healthy controls (*p* = 0.002). Yet, cfDNA did not differ between the healthy control and PAD cohort (median H: 0.28 RU, PAD: 0.31 RU, AAA: 0.44 RU). Among the investigated neutrophil and NET parameters (Table 6), MPO and citH3 were markedly correlated (*r* = 0.387). MPO and citH3 also showed strong correlations with circulating cfDNA (*r* = 0.507 and *r* = 0.599, respectively) but only weak correlations with neutrophil counts (*r* = 0.333 and *r* = 0.211, respectively).

### 3.3. Blood Lipid Levels Show an Association with Circulating NET Markers

**Figure 2:**
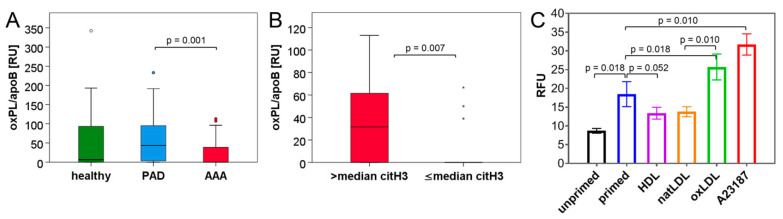
Assessment of oxPL/apoB plasma levels in healthy donors and CVD patients and regulation of NET formation by oxLDL in primed neutrophils. (**A**) Boxplots illustrate the plasma levels of oxPL/apoB as determined by ELISA in healthy donors and patients with PAD or AAA. (**B**) Plasma levels of oxPL/apoB are depicted for AAA patients with citH3 concentrations above or below (≤) the median. Statistical significance was determined by Mann–Whitney U test. (**C**) NET formation of primed neutrophils is enhanced by oxLDL but not by natLDL or HDL. Neutrophils were isolated from the peripheral blood of healthy donors and were left untreated or primed for 30 min with 5 µM A23187. Neutrophils were then washed and subsequently exposed to 100 µg/mL oxLDL, natLDL or HDL (or again to 5 µM A23187) as indicated. DNA release (mean ± SEM) was quantitated after 90 min in relative fluorescence units (RFU). Statistical analysis is based on paired *t*-test (*n* = 8 assays).

The analysis of blood lipids (Table 7) revealed the highest levels of triglycerides in AAA patients, which significantly exceeded the levels of PAD patients (median H: 123 mg/dL, PAD: 85 mg/dL, AAA: 136 mg/dL). Conversely, high-density lipoprotein (HDL) was lowest in the AAA group (median H: 55 mg/dL, PAD: 54 mg/dL, AAA: 49 mg/dL) at a significance level of *p* = 0.024 compared to healthy individuals and *p* = 0.051 compared to PAD patients. In line with the higher frequency of statin prescription in the PAD and AAA group, low-density lipoprotein (LDL) and total cholesterol were significantly lower in the CVD cohorts as compared to the healthy control collective (median LDL in H: 122 mg/dL, PAD: 61 mg/dL, AAA: 86 mg/dL). Yet, the levels of oxidized phospholipids in association with apolipoprotein B-100 (oxPL/apoB) deviated from total LDL levels (Figure 2A, Table 5); PAD patients showed the highest values (median 43 RU), while AAA patients and healthy controls did not differ significantly (0–6.33 RU).

When we proceeded to investigate the potential correlations between circulating NET biomarkers and blood lipids across groups (Table 6), only the NET parameter citH3 correlated weakly and positively with triglycerides (*r* = 0.291) and the total cholesterol to HDL ratio (*r* = 0.215). With respect to the combined data of all three cohorts, oxPL/apoB did not correlate with the explored NET or neutrophil markers. Yet, when the analysis was confined to the AAA collective (Table 8), correlations between MPO, citH3 and cfDNA blood levels were particularly strong (*r* ≥ 0.560), and the NET parameter citH3 was significantly associated with oxLDL (oxPL/apoB) (*r* = 0.470). A correlation between citH3 and oxLDL was not observed for PAD patients (data not shown). In line with these results, when AAA patients were grouped into citH3^low^ (≤median) and citH3^high^ (>median) individuals (Figure 2B), the patients with elevated levels of citH3 had significantly higher blood concentrations of oxPL/apoB (median 32 RU, IQR 75 RU versus 0 RU, IQR 0 RU, *p* = 0.007).

### 3.4. NET Induction Is Promoted by oxLDL but Unaffected by HDL In Vitro

OxLDL has previously been suggested to enhance the induction of NETs triggered by other stimuli. Yet, HDL has not been investigated in this context [28]. Therefore, we aimed to compare the effects of these lipoprotein particles on NET formation in a DNA release assay. Based on an experimental design comparable to Obama et al. [28], isolated human neutrophils were either left unprimed or stimulated for 30 min with 5 µM of the calcium ionophore A23187. After a washing step, cells were incubated with 100 µg/mL oxLDL, native LDL or HDL for 90 min. The release of DNA into the culture supernatant was monitored using the incorporation of a fluorescent dye as a quantitative measure of NET formation (Figure 2C). In this setup, native LDL and HDL showed a trend to decrease DNA release by primed neutrophils (mean 18.5 RFU, natLDL: 13.8 RFU, HDL: 13.4 RFU), while oxLDL significantly enhanced the A23187-primed NET formation (25.7 RFU). In contrast, no effect of oxLDL was observed for unprimed neutrophils (data not shown).

## 4. Discussion

Our study evaluated the interrelation of lipoprotein particles with NET parameters in patients with abdominal aortic aneurysm as compared to peripheral artery occlusive disease and healthy controls. While both atherosclerosis and AAA have been associated with NET formation, NETs seem to particularly accumulate in the intraluminal thrombus and promote the inflammatory response in the aneurysm wall [13,19,20]. Since it was shown that oxLDL is able to enhance NET stimulation [28], we wanted to investigate and compare the association of circulating oxPL/apoB and NET components in patients with distinct types of cardiovascular disease.

As we have recently shown, there is evidence for increased neutrophil numbers and neutrophil activation in AAA patients [35]. Myeloperoxidase is released upon neutrophil degranulation and is an abundant component of NETs [10]. In this study, we found that MPO is significantly elevated in AAA patients, not only in comparison to the healthy donors, but also compared to PAD patients with cardiovascular disease (Figure 1A, Table 5). Since histone 3 is citrullinated in the process of NET formation [8,9], it is considered a more specific NET marker than MPO. Similar to MPO, the highest circulating levels of citH3 were recorded in the AAA group (Figure 1B, Table 5), thus indicating that NETs might play a more prominent part in the pathogenesis of AAA as compared to PAD. Furthermore, the fact that citH3 and MPO correlated with circulating nuclear DNA in plasma suggests that a substantial fraction of cell-free DNA in blood may originate from NETs (Table 6).

With respect to the effect of blood lipids on NET release, in vivo evidence is scarce. Hence, this study addressed the potential association of dyslipidemia and the propensity of NET formation in CVD patients. When investigating the different types of blood lipids in the three study cohorts, we found that indeed, the healthy control group had the highest levels of LDL and total cholesterol. This is likely attributed to the fact that only 17% of the healthy participants received lipid-lowering drugs, as compared to 90–95% in the AAA and PAD group (Table 3). Yet, serum levels of oxPL/apoB were significantly elevated in PAD patients (Figure 2A) but did not differ between healthy controls and the AAA collective. This might relate to a lower occurrence of lipid peroxidation in AAA patients, or a more pronounced retention of oxidized phospholipids at the aneurysm site. Despite the low circulating levels of oxPL/apoB, a significant correlation between oxPL/apoB and the NET marker citH3 was detected for the AAA group. This association was not apparent in the combined data set of all study groups or for the separate PAD cohort (data not shown). Furthermore, AAA patients with elevated (>median) levels of citH3 had significantly higher blood concentrations of oxPL/apoB than citH3^low^ AAA individuals (Figure 2B).

These observations would support the hypothesis that oxLDL is able to induce or enhance NET formation, in particular in the context of AAA disease. Of note, a negative correlation between the cardiovascular protective opponent of oxLDL, namely HDL, and the NET biomarker levels was not apparent from our clinical samples.

In order to investigate the relation between distinct blood lipids and NET formation in more detail, we performed an in vitro DNA release assay with human isolated neutrophils. After brief stimulation with a calcium ionophore, the cells were exposed to lipoprotein particles. The experimental setup was comparable to the studies of Awasthi et al. [27] and Obama et al. [28], where HL-60-derived neutrophils underwent short pre-incubation with a known NET stimulus (PMA), followed by treatment with 20 µg/mL native LDL (natLDL) or oxLDL. In these studies, oxLDL but not natLDL was able to enhance NET induction (3-fold). Of note, the pre-stimulation of neutrophils was required for the oxLDL effect. Comparably, in our experimental design, the addition of oxLDL (100 µg/mL) almost doubled the NET formation of neutrophils pre-primed by A23187. OxLDL addition to unprimed neutrophils did not result in DNA release beyond background levels (data not shown). We used a NET stimulus distinct from Obama et al., and additionally tested the effect of HDL and natLDL in this setup. In contrast to oxLDL, a weak inhibitory effect of HDL and natLDL on NET release was observed, which did not reach statistical significance. Thus, in line with the in vivo result (the lack of association between HDL and citH3 in blood of CVD patients), the in vitro analyses did not detect a substantial functional impact of HDL on NET formation. We further tried to reverse the experimental design, either by pre-incubating neutrophils with blood lipids and then stimulating NET induction, or by concomitantly administering both agonists. However, these approaches did not reveal any effects of oxLDL or HDL on NET formation (data not shown).

## 5. Conclusions

In this investigation, we found that neutrophil activation and NET parameters are higher in blood of AAA than PAD patients and that the serum concentration of oxPL/apoB in AAA patients is associated with NET markers. This supports previous findings on NETs in the pathogenesis of AAA [17,18,19] and indicates that NET formation might be more prominent in AAA than in other atherosclerotic cardiovascular diseases—possibly owing to the close association between NETs and the intraluminal thrombus in AAA [19,20]. Furthermore, oxLDL may be functionally connected to the NET release in AAA. Based on the in vitro results, we conclude that HDL has little (if any) impact on NET formation, while the effect of oxLDL depends on the priming state of the neutrophil: if the neutrophil is pre-actived, oxLDL may further enhance the NET release, a setting that may likely occur in vivo in the context of AAA.

## 6. Limitations of the Study

While this is the first cross-sectional study to compare levels of blood lipids and NET parameters between distinct types of cardiovascular disease (i.e., closely matched cohorts of AAA and PAD), the findings are primarily based on correlation analyses. Hence, the conclusions are limited to statistically significant associations. Proof for an in vivo mechanistic link between oxLDL and NET induction in AAA is not provided by the investigation of clinical samples.

Similarly, the source of circulating NET components in CVD patients cannot be ascertained, as systemic levels are measured. However, with respect to AAA patients, we have previously shown that citH3 levels in circulation drop significantly after surgical AAA repair [20], which strongly supports the aneurysm as the site of origin for citrullinated histones.

Furthermore, the in vitro tests were designed to go beyond previous reports and provide the first evidence that HDL (as opposed to oxLDL) has no or little impact on NET formation. While this is in line with the in vivo results showing a correlation between oxLDL but not HDL and circulating citH3 in AAA patients, the translation of the in vitro findings to in vivo is limited.

## Figures and Tables

**Table 1:** Demographics of study collectives.

Parameter	Healthy (*n* = 29)	PAD (*n* = 40)	AAA (*n* = 40)	*p*-Value (Groups)
	*n* (%)			
Sex (male)	26 (89.7%)	32 (80.0%)	32 (80.0%)	0.336 (H/AAA) 0.336 (H/PAD) 1.000 (PAD/AAA)
Smoker				
*never*	9 (31.0%)	3 (7.5%)	3 (7.5%)	0.021 (H/AAA)
*past*	14 (48.3%)	24 (60.0%)	20 (50.0%)	0.036 (H/PAD)
*current*	6 (20.7%)	13 (32.5%)	17 (42.5%)	0.639 (PAD/AAA)
	median (interquartile range)			
Age (years)	66.6 (12.0)	72.7 (13.0)	71.8 (13.0)	0.123 (H/AAA) 0.243 (H/PAD) 0.630 (PAD/AAA)
Body mass index	26.5 (5.3)	25.9 (4.1)	27.3 (5.6)	0.585 (H/AAA) 0.375 (H/PAD) 0.131 (PAD/AAA)
Pack years	25.0 (31.0)	37.0 (49.5)	40.0 (30.0)	0.066 (H/AAA) 0.073 (H/PAD) 0.942 (PAD/AAA)

*p*-values refer to group comparisons between the healthy (H), abdominal aortic aneurysm (AAA) and peripheral artery occlusive disease (PAD) collectives by Chi square test or Fisher’s exact test for expected frequencies < 5 (categorical variables) or Mann–Whitney U test (metric variables).

**Table 2:** Comorbidities of study collectives, *n* (%).

Parameter	Healthy (*n* = 29)	PAD (*n* = 40)	AAA (*n* = 40)	*p*-Value (Groups)
Hypertension	15 (51.7%)	36 (90.0%)	37 (92.5%)	<0.001 (H/AAA) <0.001 (H/PAD) 1.000 (PAD/AAA)
Hyperlipidemia	8 (27.6%)	37 (92.5%)	35 (87.5%)	<0.001 (H/AAA) <0.001 (H/PAD) 0.712 (PAD/AAA)
PAD	0 (0%)	40 (100%)	0 (0%)	1.000 (H/AAA) <0.001 (H/PAD) <0.001 (PAD/AAA)
Coronary heart disease	0 (0%)	17 (42.5%)	20 (50.0%)	<0.001 (H/AAA) <0.001 (H/PAD) 0.501 (PAD/AAA)
Myocardial infarction	0 (0%)	9 (22.5%)	13 (32.5%) *	0.002 (H/AAA) 0.008 (H/PAD) 0.339 (PAD/AAA)
Stent	0 (0%)	24 (60.0%)	9 (22.5%) *	0.014 (H/AAA) <0.001 (H/PAD) 0.002 (PAD/AAA)
Coronary artery bypass graft	0 (0%)	3 (7.5%)	2 (5.0%)	0.506 (H/AAA) 0.258 (H/PAD) 1.000 (PAD/AAA)
Diabetes mellitus	3 (10.3%)	11 (27.5%)	12 (30.0%)	0.051 (H/AAA) 0.080 (H/PAD) 0.805 (PAD/AAA)
Nephropathy	3 (10.3%)	10 (25.0%)	16 (40.0%)	0.006 (H/AAA) 0.124 (H/PAD) 0.152 (PAD/AAA)
Chronic obstructive pulmonary disease	1 (3.4%)	13 (32.5%)	8 (20.0%)	0.069 (H/AAA) 0.003 (H/PAD) 0.204 (PAD/AAA)
Tumor (past >1 year)	5 (17.2%)	5 (12.5%)	6 (15.0%)	1.000 (H/AAA) 0.732 (H/PAD) 0.745 (PAD/AAA)

*p*-values refer to group comparisons between the healthy (H), abdominal aortic aneurysm (AAA) and peripheral artery occlusive disease (PAD) collectives by Chi square test or Fisher’s exact test for expected frequencies < 5. * indicates a missing entry for 1 AAA patient.

**Table 3:** Medication of study collectives, *n* (%).

Medication	Healthy (*n* = 29)	PAD (*n* = 40)	AAA (*n*= 40)	*p*-Value (Groups)
Acetylsalicylic acid (Thrombo ASS)	5 (17.2%)	33 (82.5%)	35 (87.5%)	<0.001 (H/AAA) <0.001 (H/PAD) 0.531 (PAD/AAA)
P2Y12 antagonist (Clopidogrel)	1 (3.4%)	8 (20.0%)	5 (12.5%)	0.389 (H/AAA) 0.069 (H/PAD) 0.363 (PAD/AAA)
Vitamin K antagonist	1 (3.4%)	3 (7.5%)	3 (7.5%)	0.634 (H/AAA) 0.634 (H/PAD) 1.000 (PAD/AAA)
Xa inhibitor	1 (3.4%)	2 (5.0%)	0 (0.0%)	0.420 (H/AAA) 1.000 (H/PAD) 0.494 (PAD/AAA)
Antihypertensive therapy	15 (51.7%)	33 (82.5%)	37 (92.5%)	<0.001 (H/AAA) 0.006 (H/PAD) 0.176 (PAD/AAA)
*ACE inhibitor*	4 (13.8%)	12 (30.0%)	17 (42.5%)	0.011 (H/AAA) 0.115 (H/PAD) 0.245 (PAD/AAA)
*Angiotensin receptor inhibitor*	6 (20.7%)	11 (27.5%)	14 (35.0%)	0.196 (H/AAA) 0.517 (H/PAD) 0.469 (PAD/AAA)
*Beta blocker*	7 (24.1%)	20 (50.0%)	25 (62.5%)	0.002 (H/AAA) 0.030 (H/PAD) 0.260 (PAD/AAA)
*Ca^2+^ channel blocker*	4 (13.8%)	9 (22.5%)	6 (15.0%)	1.000 (H/AAA) 0.361 (H/PAD) 0.390 (PAD/AAA)
*Diuretic*	4 (13.8%)	11 (27.5%)	14 (35.0%)	0.048 (H/AAA) 0.173 (H/PAD) 0.469 (PAD/AAA)
Lipid-lowering agents(type)	5 (17.2%) (80% statins; 20% drug mix)	38 (95.0%) (100% statins)	36 (90.0%) (91.7% statins; 8.3% drug mix)	<0.001 (H/AAA) <0.001 (H/PAD) 0.675 (PAD/AAA)
Antidiabetic medication	3 (10.3%)	8 (20.0%)	7 (17.5%)	0.502 (H/AAA) 0.336 (H/PAD) 0.775 (PAD/AAA)
*Insulin*	0 (0.0%)	5 (12.5%)	1 (2.5%)	1.000 (H/AAA) 0.069 (H/PAD) 0.201 (PAD/AAA)
*Metformin*	3 (10.3%)	5 (12.5%)	6 (15.0%)	0.724 (H/AAA) 1.000 (H/PAD) 0.745 (PAD/AAA)
Bronchodilatators	2 (6.9%)	9 (22.5%)	11 (27.5%)	0.031 (H/AAA) 0.104 (H/PAD) 0.606 (PAD/AAA)

*p*-values refer to group comparisons between the healthy (H), abdominal aortic aneurysm (AAA) and peripheral artery occlusive disease (PAD) collectives by Chi square test or Fisher’s exact test for expected frequencies < 5.

**Table 4:** Routine blood parameters of study collectives, median (IQR).

Parameter	Healthy (*n* = 29)	PAD (*n* = 40)	AAA (*n*= 40)	*p*-Value (Groups)
Leukocytes (10^3^/µL)	5.60 (1.80)	7.16 (2.70)	6.35 (2.70)	0.034 (H/AAA) <0.001 (H/PAD) 0.029 (PAD/AAA)
Neutrophils (10^3^/µL)	3.00 (1.15)	4.52 (1.96)	4.00 (1.97)	0.016 (H/AAA) <0.001 (H/PAD) 0.023 (PAD/AAA)
D-dimer (µg/mL)	0.38 (0.50) *	0.75 (0.68) ^§^	1.26 (1.13) ^$^	<0.001 (H/AAA) 0.008 (H/PAD) 0.007 (PAD/AAA)
C-reactive protein (mg/dL)	0.24 (0.42)	0.21 (0.32)	0.27 (0.42) ^#^	0.678 (H/AAA) 0.515 (H/PAD) 0.229 (PAD/AAA)

*p*-values refer to group comparisons between the healthy (H), abdominal aortic aneurysm (AAA) and peripheral artery occlusive disease (PAD) collectives by Mann–Whitney U test. * *n* = 27, ^§^ *n* = 36, ^$^ *n* = 35, ^#^ *n* = 39.

**Table 5:** Blood levels of explorative parameters for study collectives, median (IQR).

Parameter	Healthy (*n* = 29)	PAD (*n* = 40)	AAA (*n*= 40)	*p*-Value (Groups)
citH3 (ng/mL)	292.3 (83.6)	296.4 (221.7)	353.2 (263.3) *	0.046 (H/AAA) 0.734 (H/PAD) 0.070 (PAD/AAA)
MPO (ng/mL)	7.51 (3.20)	12.11 (5.60)	14.47 (11.74)	<0.001 (H/AAA) <0.001 (H/PAD) 0.021 (PAD/AAA)
cfDNA (RU)	0.277 (0.217)	0.311 (0.190) ^§^	0.437 (0.737) ^§^	0.002 (H/AAA) 1.000 (H/PAD) 0.003 (PAD/AAA)
oxPL/apoB (RU)	6.33 (104.08)	43.42 (92.50)	0.00 (39.00) ^#^	0.197 (H/AAA) 0.181 (H/PAD) 0.001 (PAD/AAA)

*p*-values refer to group comparisons between the healthy (H), abdominal aortic aneurysm (AAA) and peripheral artery occlusive disease (PAD) collectives by Mann–Whitney U test. cfDNA, cell-free DNA; citH3, citrullinated histone H3; MPO, myeloperoxidase; oxPL/apoB, oxidized phospholipid/apolipoprotein B-100 complexes; RU, relative units; * *n* = 38, ^§^ *n* = 30, ^#^ *n* = 39.

**Table 6:** Parameter correlations for all study groups, Spearman test.

Parameter 1	Parameter 2	*r*	*p*	*n*
citH3 (ng/mL)	Neutrophils (10^3^/µL)	0.211	0.029	107
	MPO (ng/mL)	0.387	<0.001	107
	cfDNA (RU)	0.599	<0.001	89
	Triglycerides (mg/dL)	0.291	0.002	106
	Total cholesterol/HDL ratio	0.215	0.027	106
MPO (ng/mL)	Neutrophils (10^3^/µL)	0.333	<0.001	109
	cfDNA (RU)	0.507	<0.001	89
	D-dimer (µg/mL)	0.475	<0.001	98
cfDNA (RU)	Triglycerides (mg/dL)	0.257	0.016	88
oxPL/apoB (RU)	Lipoprotein (a) (nmol/L)	0.314	0.001	106

cfDNA, cell-free DNA; citH3, citrullinated histone H3; MPO, myeloperoxidase; oxPL/apoB, oxidized phospholipid/apolipoprotein B-100 complexes; RU, relative units.

**Table 7:** Lipid profile of study collectives, median (IQR).

Parameter	Healthy (*n* = 29)	PAD (*n* = 40)	AAA (*n*= 39)	*p*-Value (Groups)
Triglycerides (mg/dL)	123.0 (82.0)	85.0 (63.0)	136.0 (124.0) *	0.097 (H/AAA) 0.256 (H/PAD) 0.001 (PAD/AAA)
Total cholesterol (mg/dL)	200.0 (36.0)	141.5 (43.0)	162.0 (79.0)	0.003 (H/AAA) <0.001 (H/PAD) 0.031 (PAD/AAA)
HDL cholesterol (mg/dL)	55.0 (22.0)	54.0 (28.0)	49.0 (16.0)	0.024 (H/AAA) 0.559 (H/PAD) 0.051 (PAD/AAA)
LDL cholesterol (mg/dL)—calculated	122.2 (42.1)	60.5 (32.5)	85.6 (72.8)	0.007 (H/AAA) <0.001 (H/PAD) 0.056 (PAD/AAA)
Total cholesterol/HDL ratio	3.9 (2.2)	2.7 (1.4)	3.8 (1.8)	0.742 (H/AAA) 0.001 (H/PAD) 0.001 (PAD/AAA)
LDL/HDL ratio	2.4 (1.7)	1.3 (0.8)	1.7 (1.6)	0.210 (H/AAA) <0.001 (H/PAD) 0.013 (PAD/AAA)
Lipoprotein (a) (nmol/L)	13.0 (33.0)	46.5 (123.0)	18.0 (65.0) ^#^	0.394 (H/AAA) 0.051 (H/PAD) 0.177 (PAD/AAA)

*p*-values refer to group comparisons between the healthy (H), abdominal aortic aneurysm (AAA) and peripheral artery occlusive disease (PAD) collectives by Mann–Whitney U test. HDL, high-density lipoprotein; LDL, low-density lipoprotein; * *n* = 39, ^#^ *n* = 38.

**Table 8:** Parameter correlations for the AAA collective, Spearman test.

Parameter 1	Parameter 2	*r*	*p*	*n*
oxPL/apoB (RU)	citH3 (ng/mL)	0.470	0.003	37
citH3 (ng/mL)	Neutrophils (10^3^/µL)	0.363	0.025	38
	cfDNA (RU)	0.680	<0.001	30
	MPO (ng/mL)	0.560	<0.001	38
MPO (ng/mL)	cfDNA (RU)	0.566	0.001	30

cfDNA, cell-free DNA; citH3, citrullinated histone H3; MPO, myeloperoxidase; oxPL/apoB, oxidized phospholipid/apolipoprotein B-100 complexes; RU, relative units.

## Data Availability

The data presented in this study are available on request from the corresponding author.

## Abbreviations

AAA, abdominal aortic aneurysm; cfDNA, cell-free DNA; citH3, citrullinated histone H3; CTA, computed tomography angiography; CTAD, citrate, theophylline, adenosine and dipyridamole; CVD, cardiovascular disease; ELISA, enzyme-linked immunosorbent assay; H, healthy controls; HBSS, Hank’s balanced salt solution; HDL, high-density lipoprotein; IQR, interquartile range; LDL, low-density lipoprotein; LPS, lipopolysaccharide; MPO, myeloperoxidase; natLDL, native, low-density lipoprotein; NET, neutrophil extracellular trap; oxLDL, oxidized, low-density lipoprotein; oxPL/apoB, oxidized phosholipid in association with apolipoprotein B-100; PAD, peripheral artery occlusive disease; PMA, phorbol 12-myristate 13-acetate; qPCR, quantitative polymerase chain reaction; RFU, relative fluorescence units; RU, relative units
